# The p53^R172H^ Mutant Does Not Enhance Hepatocellular Carcinoma Development and Progression

**DOI:** 10.1371/journal.pone.0123816

**Published:** 2015-04-17

**Authors:** Leanne G. Ahronian, David R. Driscoll, David S. Klimstra, Brian C. Lewis

**Affiliations:** 1 Department of Molecular, Cell and Cancer Biology, University of Massachusetts Medical School, Worcester, Massachusetts, United States of America; 2 Department of Pathology, Memorial Sloan-Kettering Cancer Center, New York, New York, United States of America; 3 Program in Molecular Medicine, University of Massachusetts Medical School, Worcester, Massachusetts, United States of America; 4 Cancer Center, University of Massachusetts Medical School, Worcester, Massachusetts, United States of America; University of Hong Kong, HONG KONG

## Abstract

Hepatocellular carcinoma is a highly deadly malignancy, accounting for approximately 800,000 deaths worldwide every year. Mutation of the p53 tumor suppressor gene is a common genetic change in HCC, present in 30% of cases. p53^R175H^ (corresponding to p53^R172H^ in mice) is a hotspot for mutation that demonstrates “prometastatic” gain-of-function in other cancer models. Since the frequency of p53 mutation increases with tumor grade in HCC, we hypothesized that p53^R172H^ is a gain-of-function mutation in HCC that contributes to a decrease in tumor-free survival and an increase in metastasis. In an HCC mouse model, we found that p53^R172H/flox^ mice do not have decreased survival, increased tumor incidence, or increased metastasis, relative to p53^flox/flox^ littermates. Analysis of cell lines derived from both genotypes indicated that there are no differences in anchorage-independent growth and cell migration. However, shRNA-mediated knockdown of mutant p53 in p53^R172H^-expressing HCC cell lines resulted in decreased cell migration and anchorage-independent growth. Thus, although p53 mutant-expressing cells and tumors do not have enhanced properties relative to their p53 null counterparts, p53^R172H^-expressing HCC cells depend on this mutant for their transformation. p53 mutants have been previously shown to bind and inhibit the p53 family proteins p63 and p73. Interestingly, we find that the levels of p63 and p73 target genes are similar in p53 mutant and p53 null HCC cells. These data suggest that pathways regulated by these p53 family members are similarly impacted by p53^R172H^ in mutant expressing cells, and by alternate mechanisms in p53 null cells, resulting in equivalent phenotypes. Consistent with this, we find that p53 null HCC cell lines display lower levels of the TA isoforms of p63 and p73 and higher levels of ΔNp63. Taken together these data point to the importance of p63 and p73 in constraining HCC progression.

## Introduction

Liver cancer accounts for approximately 800,000 deaths annually and up to 85% of these cancers are hepatocellular carcinoma (HCC) [1]. Curative treatments for HCC are restricted to surgical resection of the tumor, or liver transplantation. Unfortunately, as few as 30% of patients are eligible for resection or transplant due to the presence of extensive liver disease, invasive HCC, or metastasis [2,3]. Moreover, relapse rates post-resection are over 60%, suggesting the presence of undetected disease dissemination at the time of surgery [4]. At present, there are no curative options for patients with unresectable disease. These patients are commonly treated with Sorafenib, which extends survival by 2.8 months [5]. Therefore, understanding the molecular mechanisms underlying HCC dissemination is of great importance for improving the prognosis for HCC patients.

Point mutations in the *TP53* tumor suppressor gene occur at a high frequency in many tumor types [6]. In HCC, *TP53* gene mutation is observed in over 30% of cases [7]. Interestingly, *TP53* mutations are absent in hepatic adenomas, while their frequency increases with tumor grade and differentiation status, occurring in 54% of poorly differentiated HCCs [8,9]. Indeed, *TP53* mutations are associated with a higher rate of relapse and decreased overall survival in HCC [7,10]. Furthermore, in a non-metastatic HCC mouse model, deletion of *Trp53* resulted in tumors with more aggressive histology and increased metastasis to the lungs [11]. Together, these findings suggest a specific role for p53 inactivation in promoting HCC progression.

Some p53 missense mutations have been found to exert both dominant negative and gain-of-function effects [12]. One particular mutation, p53^R172H^, which corresponds to human p53 hotspot R175H, has been shown to inhibit wild-type p53 function [13–15]. Aside from inactivating the wild-type protein, p53^R172H^ also displays gain-of-function properties in breast and pancreatic cancer, with phenotypes including increased tumor initiation, invasion, and metastasis relative to p53 null controls [16–19]. Moreover, mice bearing a single knock-in *LSL-p53* ^*R172H*^ allele developed more carcinomas than p53 null counterparts, consistent with gain-of-function properties of the mutant protein [20]. Finally, tumors expressing p53^R172H^ were more metastatic than tumors deleted for p53 [20,21].

Whether p53 mutants display gain-of-function activity in liver cancer is unclear. A prior study found that overexpression of several p53 mutants in HCC cell lines decreased apoptosis in response to stress [22]. In another study, ectopic expression of an aflatoxin-induced p53^R249S^ mutant did not confer any growth benefit to an HCC cell line. However, in an HCC cell line with endogenous expression of p53^R249S^, p53 knockdown decreased proliferation and increased cell death [23]. These data demonstrate that the response of HCC cell lines to mutant p53 may vary depending on endogenous or exogenous expression of these mutants. Additionally, cell context and the type of p53 mutant may also be important factors dictating p53 gain-of-function activity in HCC.

Yet, p53 mutants do not display gain-of-function properties in every context. In a UV-induced skin carcinogenesis model, the p53^R270H^ mutant exerted dominant-negative effects on the wild-type p53 protein and enhanced tumor formation and decreased survival [24]. However, mice expressing only the p53^R270H^ mutant in the skin did not have enhanced tumor formation or decreased survival relative to animals null for p53, suggesting the absence of gain-of-function properties in this tissue [24].

The R172H mutation alters the tertiary structure of the p53 protein and is therefore classified as a structural mutant [25]. This mutant is thought to bind and inhibit the p53-related transcription factors p63 and p73, resulting in gain-of-function effects [21,26]. Expression of *TP63* and *TP73* genes can be directed from two distinct promoters, resulting in TA or ΔN isoforms [27]. In general, TA isoforms are believed to act as tumor suppressors, while the ΔN isoforms are thought to be oncogenic. Mice with heterozygous deletion of *Trp63* or *Trp73* develop spontaneous tumors with accompanying loss of heterozygosity, indicating that these transcription factors act as tumor suppressors [28]. In addition, inhibition of these other p53 family members exacerbates tumor development and progression in p53-deficient animals, suggesting that they have non-overlapping tumor suppressor functions with p53 [28,29]. Moreover, recent work demonstrated that specific deletion of the ΔN isoforms of inhibited growth of p53-deficient tumors [30].

However, unlike the pattern of *TP53* inactivation seen in HCC patient specimens, mutations or allelic losses have not been detected in the *TP63* and *TP73* genes [31,32]. Interestingly, TAp73 and TAp63 levels are increased in HCCs as compared to normal liver [31,33]. In the case of p73, higher expression levels are correlated with p53 inactivation [34,35] and poorer prognosis [36]. Yet in the mouse, concomitant deletion of *Trp53* and *Trp73* promotes HCC development [28].

Given the absence of homozygous *TP53* deletion, and because p53^R172H^ has been shown to contribute to metastasis in other mouse models, we investigated whether p53^R172H^ enhances HCC progression relative to p53 nullizygosity. Interestingly, we find that the p53^R172H^ mutant does not enhance tumor development or metastasis *in vivo*, relative to p53 deficiency. Further, cell lines derived from p53 null and p53^R172H^-expressing tumors display similar phenotypes *in vitro*. Intriguingly, we find that p53^R172H^-expressing HCC cells are dependent on the sustained expression of this mutant for their transformed phenotype. Finally, our data suggest that impairment of p63 and p73 function occurs in all of the HCC cell lines evaluated, suggesting that inhibition of these transcription factors may be a critical step in HCC pathogenesis.

## Materials and Methods

### Ethics Statement

All animal experiments were reviewed and approved by the University of Massachusetts Medical School Institutional Animal Care and Use Committee. Animals were housed in specific pathogen free facilities with abundant food and water.

### Animal Studies

*LSL-Trp53* ^*R172H*^ mice [20] were obtained and crossed with *Albumin-tva*, *p53* ^*flox/flox*^, *Albumin-cre* mice [37]. *Albumin-tva*, *LSL-Trp53* ^*R172H/flox*^, *Albumin-cre* mice were crossed to *Albumin-tva*, *Trp53* ^*flox/flox*^, *Albumin-cre* mice in order to obtain littermates for direct comparison. Three day-old pups were injected in the liver with 2x10^6^ DF1 cells producing RCAS-*PyMT* in 5μL of serum free DMEM [37]. Mice in the tumor study were divided into either survival or time point cohorts. In the survival study, a cohort of 30 injected mice were sacrificed when distress was apparent as defined by our University of Massachusetts Medical School Institutional Animal Care and Use Committee approved animal protocol (hunched posture, poor grooming, reduced activity, and altered respiratory pattern), or at the age of 9 months, whichever came first. Euthanasia was performed by lethal CO_2_ administration followed by bilateral pneumothorax, a method consistent with the recommendations of the Panel on Euthanasia of the American Veterinary Medical Association. Mice whose cause of death was likely due to tumor burden or metastatic disease were counted in the Kaplan-Meier curve, while mice that died without primary HCC were censored. Kaplan-Meier statistics were calculated using the Log-rank test. An additional cohort of mice was assigned to time point groups of 3, 6, or 8 months as they were enrolled in the study. Mice were sacrificed at these time points, or when distress was apparent. Individual tumors were counted and measured in three dimensions. Tumor volume was calculated using the formula for an ellipsoid (V = (4/3)π*L/2*W/2*H/2, where L, W and H are the length, width and height of the tumor, respectively*)*, and tumor burden/mouse was the sum of each tumor volume per animal. For both the time point and survival studies, liver and lung tissues from each mouse were harvested. Portions of fresh HCC were excised from harvested tissue in order to establish cell lines. Additionally, portions of tumor were flash frozen in liquid nitrogen for later isolation of nucleic acids and protein. Remaining liver and lung tissue from each animal were formalin-fixed and paraffin-embedded for further analysis.

### Cell culture

Tumor-derived cell lines were isolated de novo by mincing mouse tumor tissue and culturing in high-glucose DMEM with 10% fetal bovine serum (FBS) and 1% Penicillin/Streptomycin (Pen/Strep). Appropriate p53 expression was confirmed by immunoblot.

Stable p53 knockdown was performed using a pGIPZ library shRNA directed to mouse p53 (Thermo Scientific, V3LHS_646511). An empty pGIPZ plasmid was used as a control. Lentiviruses were produced in 293T cells that were transfected with plasmids containing envelope glycoprotein, required packaging sequences, and pGIPZ plasmid. Transfections were performed using Effectene Transfection Reagent (QIAGEN, 301425). HCC target cells were infected with 1 mL of lentiviral supernatant in a well of a 6-well plate in the presence of 1μg polybrene. Following infection, cells were selected in 6μg/mL puromycin before experimental analysis. To induce cell stress and p53 expression, 50nM Gemcitabine was added to growth media for 24 hours before generating protein lysates.

### Transwell migration assay

Migration of cell lines was assessed by plating 2.5x10^4^ cells on a migration insert (BD Biosciences, 354578) in serum-free DMEM. DMEM containing 10% FBS was placed in the lower chamber as a chemoattractant. After 20 hours, non-migrating cells were removed from the upper side of the membrane, and migrating cells on the lower side of the membrane were stained with Giemsa. The migrating cells per well were determined by averaging the cell numbers across 5 fields at 100X magnification. Error bars on migration assay graphs are representative of standard deviation. p-values were calculated by Student’s t-test.

### Soft agar colony assay

For soft agar colony formation experiments, a 1.4% hard agar solution was combined with 2X DMEM with 20% FBS and 2% Pen/Strep and plated onto 100mm tissue culture plates. After hardening, 1x10^5^ cells were mixed with equal parts of a 0.8% soft agar solution and 2X growth media. After the soft agar solution hardened, 8ml of 1X growth media was added to the dishes, which were incubated for 2 weeks at 37°C. Colonies were counted in 20 fields per plate at 100X magnification. Size exclusion was used to ensure that colonies had grown in the dish, and were not just cell clumps. Error bars on soft agar colony experiments are representative of standard deviation. p-values were calculated by Student’s T-test.

### Immunoblotting

Protein lysates from cell lines were generated in RIPA buffer, and concentrations were normalized by Bradford Assay. 20μg of lysate were used for each Western blot sample. 10% acrylamide gels were used for SDS-PAGE, and subsequently blotted onto PVDF. Western blots were blocked for one hour at room temperature in 7.5% nonfat dry milk in TBS. Antibodies for Western blotting were diluted in either 5% nonfat dry milk or 5% BSA. Antibodies used were: p53 antibody (1:2000, Cell Signaling 1C12), p21 antibody (1:1000, Santa Cruz sc-6246), TAp63 antibody (1:500, Biolegend 618901), ΔNp63 antibody (1:500, Biolegend 619001), Total p63 antibody (1:500 Santa Cruz, ac-8431), TAp73 antibody (1:500 Novus 24737), Total p73 antibody (1:500 Imgenex). GAPDH antibody (1:1000, Millipore, MAB374) and rabbit polyclonal β-actin antibody (1:2000, sc-1615-R).

### Immunohistochemistry

Portions of each tumor were fixed in formalin and embedded in paraffin. Tissue sections were deparaffinized with heat and rehydrated in decreasing alcohol concentrations. After rehydration, antigen retrieval was performed by microwaving slides in Antigen Unmasking Solution (Vector, H-3300). After cooling, endogenous peroxides were inactivated by adding 3% hydrogen peroxide for 10 minutes. Slides were blocked in goat serum in PBS for one hour at room temperature. Ki67 primary antibody (1:500, Abcam, ab66155) and p53 antibody (1:500, Leica Biosystems, CM5) were diluted in goat serum in PBS and incubated on slides overnight at 4°C. Biotinylated Rabbit secondary antibody was diluted in goat serum in PBS and added to the slides for one hour at room temperature. Goat serum, secondary antibody, and developing reagents were from Vector ABC kits (PK-4001). Pigment was developed in the tissues using a NovaRed Peroxidase Substrate (Vector, SK-4800). After development, slides were co-stained in hematoxylin, dehydrated, and mounted.

### p53 family target gene analysis

RNA from cell lines was isolated using Trizol, and converted to cDNA using First-Strand cDNA synthesis kit (Invitrogen, 18080–051). qRT-PCR was performed using Quanta Perfecta SYBR Green (VWR, 95072) in an ABI 7300 machine using 50ng cDNA. p63 and p73 targets were amplified using the primers listed in S1 Table.

For qPCR studies on the tumor-derived cell line panel, average C_t_ values for each cell line were determined for both the target gene and β-actin endogenous control. C_t_ values for each individual cell line were normalized to the average C_t_ value for the p53-null cell lines, so that each cell line fold change is calculated in relationship to the average of four p53^fl/fl^ cell lines. All individual lines were normalized to β-actin as an endogenous reference.

For assaying p53 knockdown cell lines, each shRNA-infected line was normalized to its respective pGIPZ-infected control, after normalizing to an endogenous reference. In each case the Comparative C_t_ Method was used to calculate fold change, where fold change = 2^-ΔΔCT^.

## Results

### p53R172H does not promote HCC development, progression, and metastasis *in vivo*

**Fig 1:**
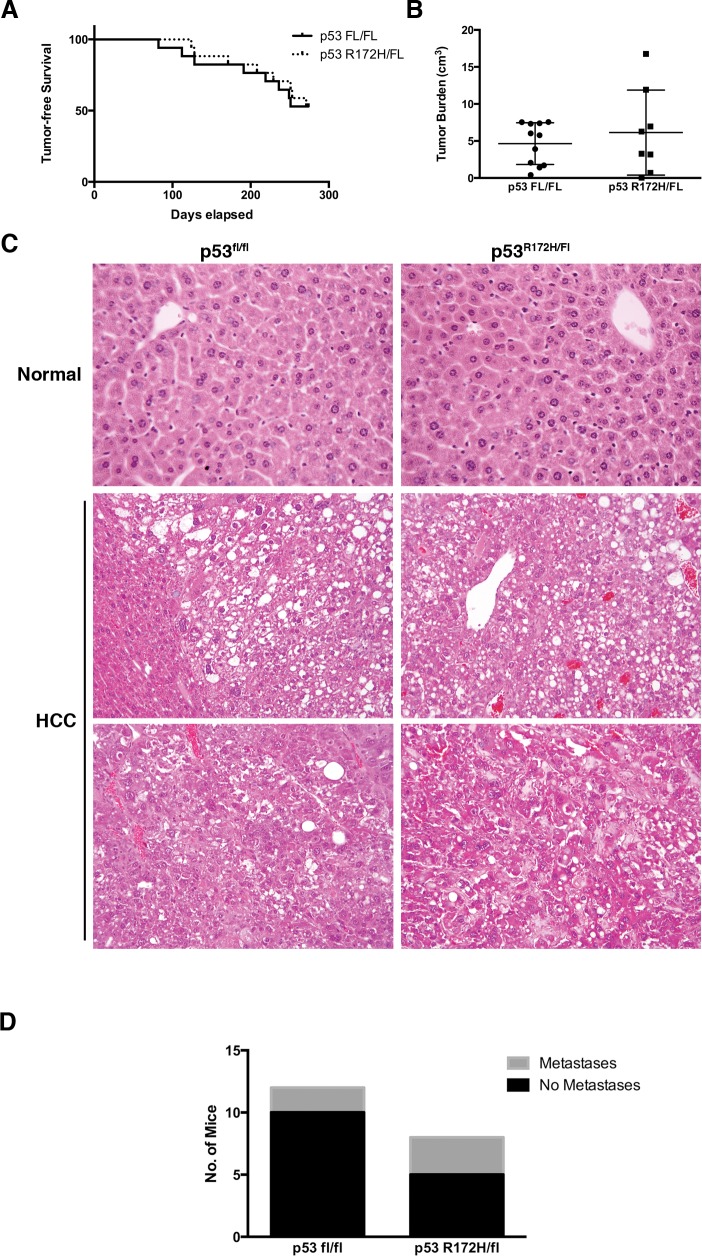
p53^R172H^ does not result in gain-of-function properties in an HCC mouse model. A) Kaplan-Meier curve displaying tumor free survival of p53^fl/fl^ and p53^R172H^ littermates. Mice were assessed over a period of 9 months and sacrificed when illness was apparent. Only mice whose death was perceived to be due to HCC were included, otherwise the mouse was censored. Censored animals are represented in the figure as tick marks on the day of death. B) Total tumor burden in the survival cohort. Individual tumor volume was calculated using the formula for an ellipsoid. All of the tumor volumes for a given mouse were summed to arrive at the tumor burden per animal. C) H&E staining demonstrating commonly seen architecture in HCC mouse model. D) Number of p53^R172H^ and p53^fl/fl^ mice with (grey) or without (black) grossly visible metastatic lesions.

To determine if p53^R172H^ exhibits gain-of-function properties in HCC, we generated *Albumin-tva*, *LSL-Trp53* ^*R172H/flox*^, *Albumin-cre* mice (hereafter referred to as p53^R172H^ mice) and *Albumin-tva*, *Trp53* ^*flox/flox*^, *Albumin-*cre (p53^fl/fl^) littermates. These mice were injected with DF1 cells producing RCAS-*PyMT* to induce HCC, as done previously [11,37]. In this model, loss of p53 function promotes metastasis, which can be enhanced through concomitant deletion of *Ink4a/Arf* [11,37]. Therefore, if the p53^R172H^ mutant has gain-of-function properties, these should be observed in this model as enhanced tumor progression. We found that the presence of the mutated p53 allele did not impact tumor-free survival (Fig 1A). Immunohistochemical staining of tumor specimens detected nuclear p53 in p53^R172H^ tumors but not p53^fl/fl^ tumors, demonstrating specific expression within the p53 mutant tumors (S1 Fig). Analysis of genomic DNA isolated from a subset of liver tumors also confirmed recombination of the LSL cassette in the p53^R172H^ tumors confirming activation of the mutant p53 allele (S1 Fig).

To ascertain if p53^R172H^ accelerated tumor onset in this model, a separate cohort of animals was euthanized at 3, 6, or 8 months of age, unless the mouse displayed signs of high tumor burden and had to be euthanized in accordance with our approved animal protocol. This cohort also did not display different rates of tumor formation or metastasis at any time point evaluated (S2 Table).

In addition to these characteristics, we assessed whether tumor burden or tumor histology differed between tumors induced in p53^R172H^ and p53^fl/fl^ mice. We found that the total tumor burden for each mouse in the survival study was not statistically different between the two groups (Fig 1B). Moreover, the tumors induced in the two groups appeared to be histologically similar. Evaluation of hematoxylin and eosin (H&E) stained tissue sections demonstrated that regions of unique HCC histology appear at similar frequencies in both groups (Fig 1C). Ki67 staining of tumor tissues also revealed that the proliferation rates are similar between tumors with or without the p53 mutant (S2 Fig). Indeed, variation between tumors within the same group was greater than variation between the two groups. In addition, p53^R172H^ and p53^fl/fl^ mice did not have significantly different rates of metastasis (Chi-Square, p = 0.2918; Fisher’s Exact Test, p = 0.3473), with 2/12 p53^fl/fl^ mice, and 3/8 p53^R172H^ mice displaying metastasis to the lungs or within the peritoneal cavity (Fig 1D and S3 Fig). Collectively, these data demonstrate that the presence of the p53^R172H^ mutant does not enhance HCC development and progression *in vivo*.

### Tumor-derived cell lines from p53^R172H^ HCCs do not display enhanced properties relative to p53^fl/fl^-derived cell lines

**Fig 2:**
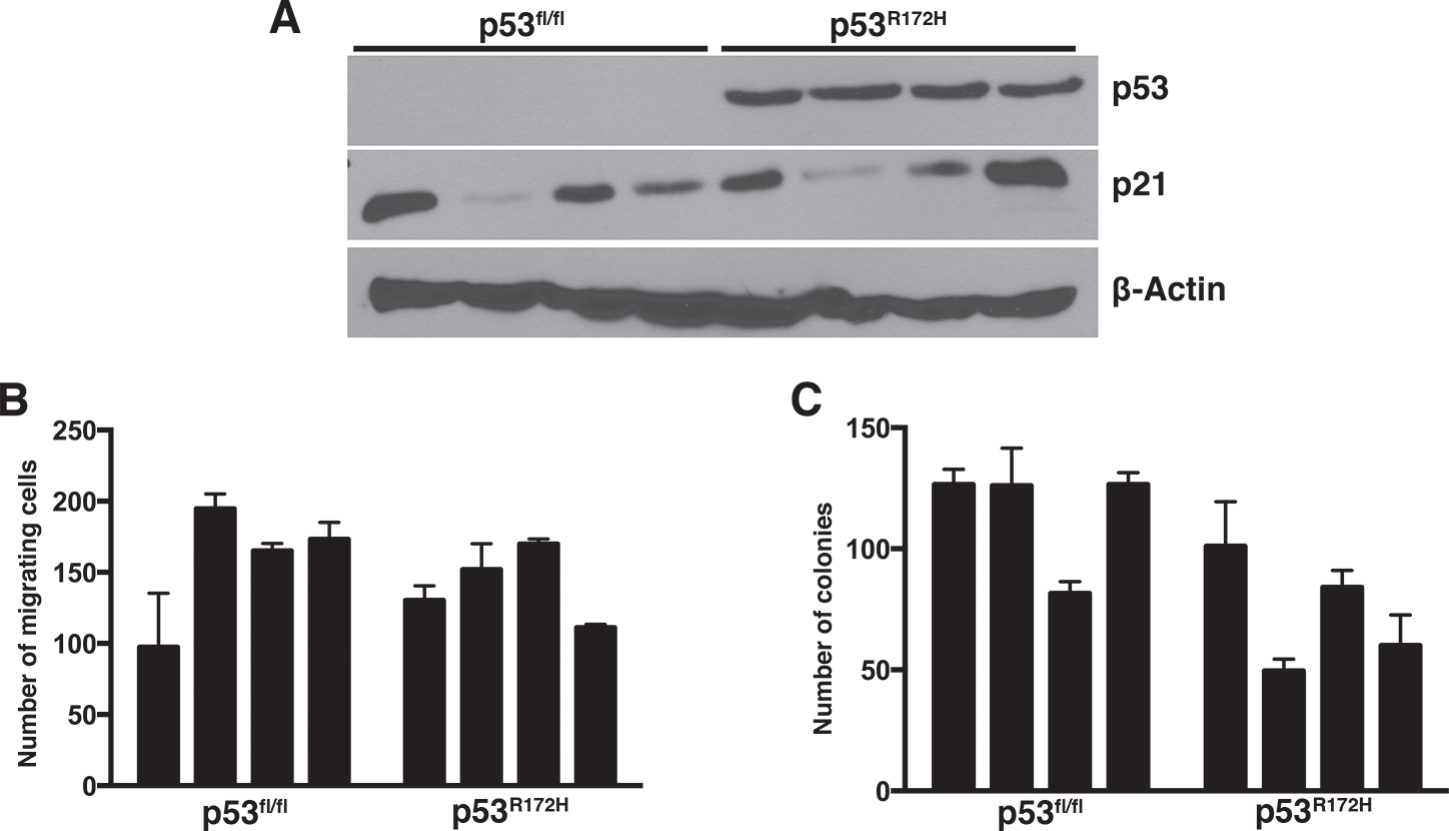
Cell lines derived from p53^R172H^-expressing HCCs do not display enhanced properties when compared to p53-null HCCs. A) Immunoblots demonstrating expression of p53 and p21 in selected mouse tumor-derived cell lines. B) Transwell migration assay of HCC cell lines derived from p53^fl/fl^ and p53^R172H^ tumors. C) Soft agar colony formation assay of HCC cell lines derived from p53^fl/fl^ and p53^R172H^ tumors.

We generated a collection of HCC cell lines isolated from tumors induced in p53 null and p53 mutant livers. Eight (8) tumor cell lines, 4 from each group, were compared to determine whether the presence of p53^R172H^ enhanced transformation-associated phenotypes *in vitro*. p53^R172H^ tumor-derived cell lines expressed p53 as expected, while p53^fl/fl^ cell lines had no detectable p53 (Fig 2A). Importantly, expression of the p53 target gene p21 was not induced in p53^R172H^ cell lines relative to p53^fl/fl^ lines following gemcitabine treatment, consistent with the absence of functional p53 protein (Fig 2A).

To ascertain whether p53^R172H^ enhances HCC cell migration, we performed transwell migration assays, and found that p53 null cell lines migrated as well as p53 mutant lines (Fig 2B). Thus, the presence of mutant p53 does not enhance this property, an outcome consistent with the similar rates of metastasis observed *in vivo* in our HCC mouse model. Similarly, we found that p53^R172H^ does not enhance anchorage-independent growth, relative to p53 nullizygosity, as measured by a soft agar colony formation assay (Fig 2C). These data are consistent with our finding of similar survival rates and tumor burden in the HCC mouse model. Thus, in combination with our *in vivo* data, our *in vitro* results suggest that the p53^R172H^ mutant does not promote an enhanced tumor phenotype in HCC.

### p53^R172H^ is required for HCC cell transformation

**Fig 3:**
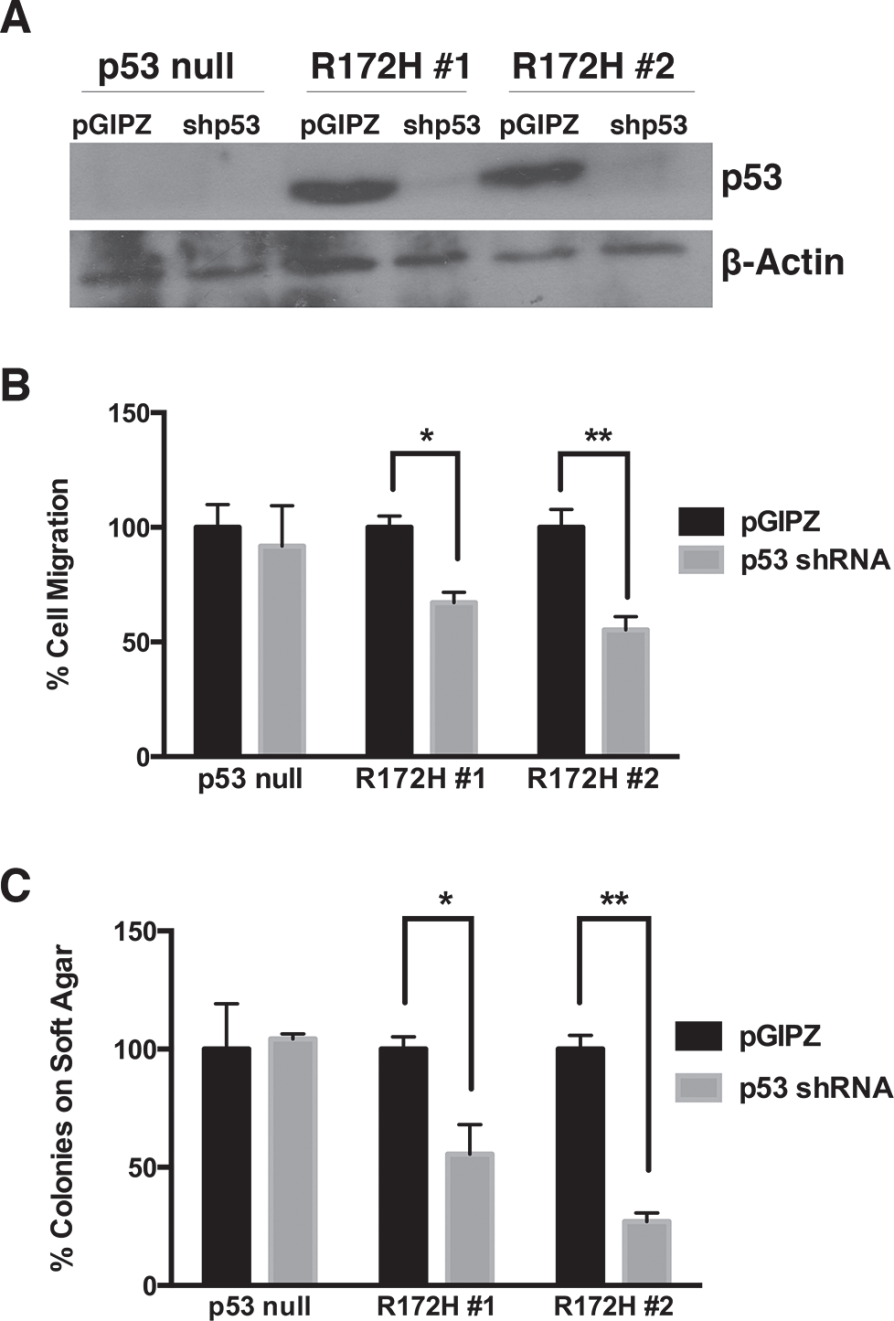
p53^R172H^ is required for transformation-related phenotypes in HCC cell lines. A) Immunoblot of p53 depicting 3 HCC cell lines infected with shRNA as compared to an empty vector control labeled pGIPZ. B) Transwell migration assay of p53 shRNA-infected HCC cell lines. A p53-null cell line was used as an experimental control. p-values, calculated by Student’s t-test, are 0.020 and 0.022 for R172H lines 1 and 2, respectively. The number of migrating cells in each pGIPZ control infection was set to 100%. C) Soft agar colony formation assay of p53 shRNA-infected HCC cell lines. A p53-null cell line was used as an experimental control. p-values, calculated by Student’s t-test are 0.044 and 0.004 for R172H lines 1 and 2, respectively. The number of colonies for each pGIPZ control infection was set to 100%.

In previous studies analyzing the gain-of-function properties of mutant p53 proteins, a hallmark experiment was the demonstration that knockdown of mutant p53 abrogated the transformed phenotype [20,22]. We therefore determined whether depletion of mutant p53 similarly impacted our HCC cell lines. We knocked down p53 in 2 cell lines derived from p53^R172H^ tumors and knockdown confirmed by immunoblotting (Fig 3A). As a control for potential off-target effects, we introduced the p53-targeting shRNA into a p53 null cell line. We observed that p53 knockdown impaired cell migration (Fig 3B) and soft agar colony formation (Fig 3C) relative to controls in cells expressing the R172H mutant, suggesting that mutant p53 is required for these phenotypes in p53^R172H^-expressing HCC cell lines. Importantly, expression of the p53-targeting shRNA in a p53 null cell line did not impact cell migration or soft agar colony formation, confirming that these effects result from suppression of mutant p53 and not off-target effects.

### p53 family transcriptional activity is similar in p53 null and p53^R172H^-expressing HCC lines

**Fig 4:**
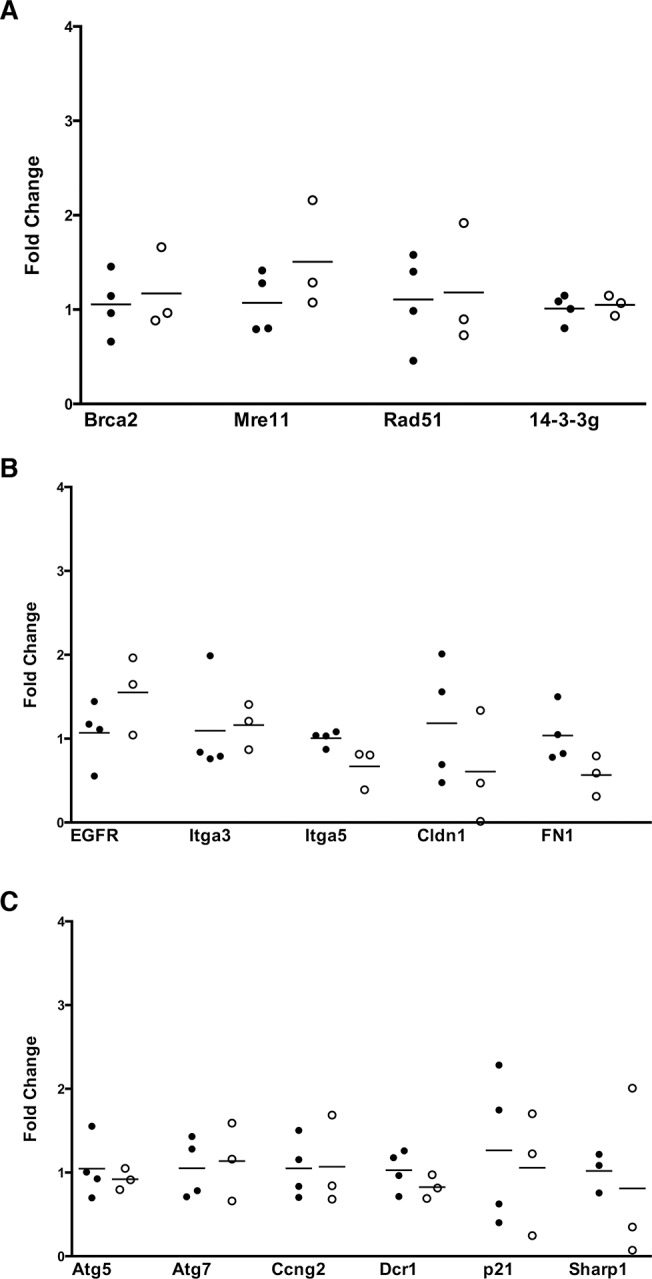
p53^R172H^ and p53^fl/fl^ HCC cell lines do not display differential expression of p53 family transcriptional targets. A) qRT-PCR of DNA damage-related targets of p53 family transcription factors. B) qRT-PCR analysis of p53 family targets related to cell signaling. C) qRT-PCR analysis of p53 family targets related to other cell processes. Dark circles indicate p53^fl/fl^ cell lines, while open circles are p53^R172H^ cell lines.

The above data suggest that while p53^R172H^ is required for transformation-associated phenotypes in HCC cells expressing the mutant, the presence of mutant p53 does not enhance these properties beyond what is observed in p53 null cell lines. This suggests, potentially, that similar pathways are impacted in both contexts. Since p53^R172H^ is known to bind to, and inhibit, other p53 family transcription factors as a mechanism of its gain-of-function properties in other cancer types, we examined the mRNA levels of a collection of genes known to be regulated by the related p63 and p73 proteins. Previously published studies have described several genes regulated by p63 and p73. We curated a collection of published target genes from such studies [38–40], and selected a subset of these genes that are involved in a range of cellular processes for characterization in our HCC cell lines. We found that the mRNA levels of p53 family target genes involved in cell signaling (Fig 4A), DNA damage (Fig 4B), and cellular processes (Fig 4C), are unchanged between p53^R172H^ and p53^fl/fl^ cell lines.

### p53 family target gene expression is increased upon knockdown of mutant p53

Tumor-derived cell lines from our mouse model containing p53^R172H^ do not have enhanced tumor properties as compared to cell lines null for p53. This could in part be attributed to similar levels of p53 family transcriptional activity as shown in Fig 4. However, upon knockdown of p53^R172H^, cell transformation properties decrease, indicating that mutant p53 is required for the phenotypes displayed in these cells (Fig 3).

**Fig 5:**
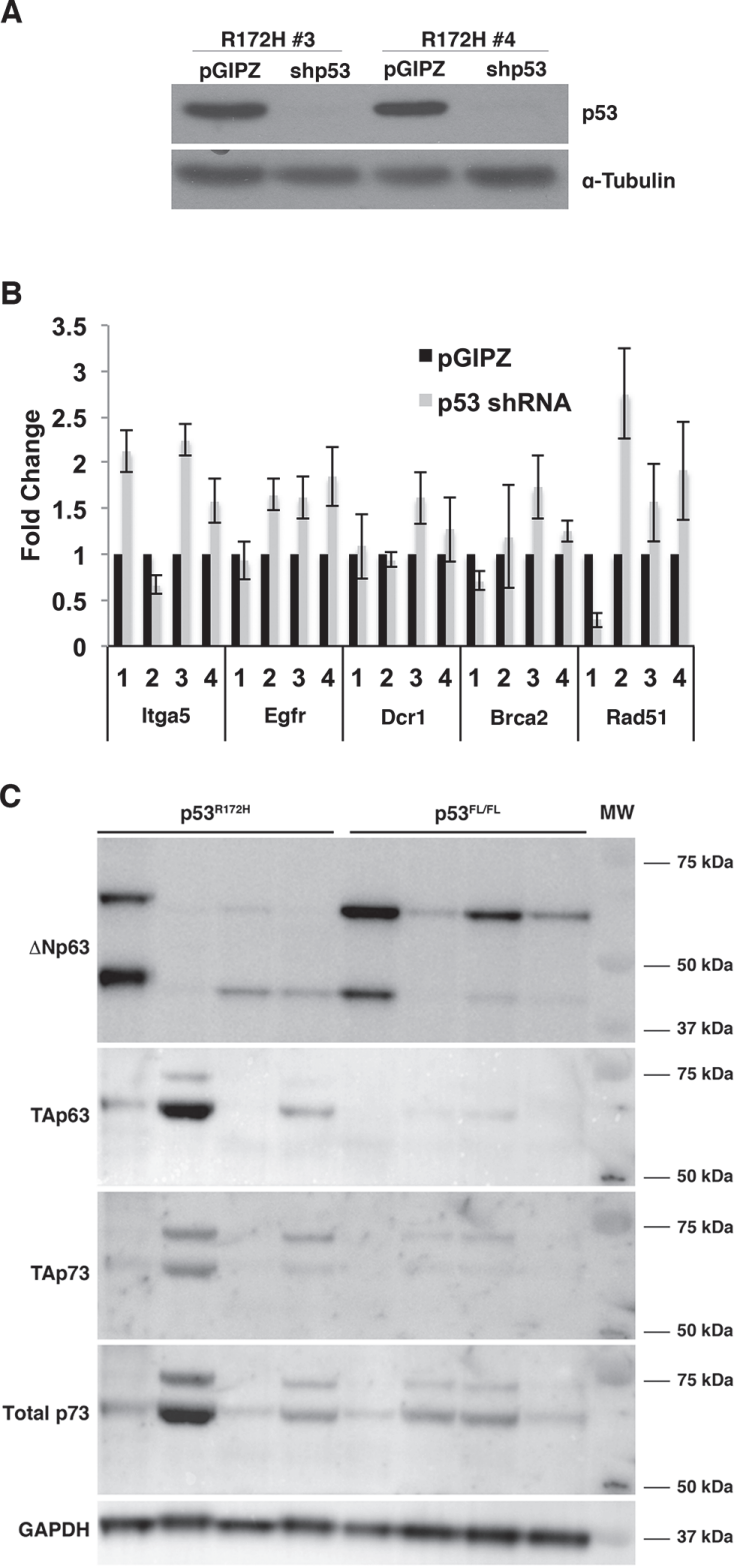
Knockdown of p53 in p53^R172H^ HCC cell lines results in increased expression of several p53 family transcriptional targets. A) Immunoblot demonstrating knockdown of p53 in cell lines derived from p53^R172H^ HCCs as compared to pGIPZ empty vector control infections. B) Quantitative RT-PCR analysis of p53 family transcriptional targets in four mouse HCC cell lines with knockdown of p53^R172H^. Ct values in each sample were normalized to β-Actin as an endogenous reference. Fold changes were calculated by normalizing all samples to the average Ct value of the cell line’s pGIPZ control. The Comparative Ct method was used for fold change calculation. C) Immunoblots demonstrating the expression of p63 and p73 isoforms in p53^fl/fl^ and p53^R172H^ HCC cell lines. Note the presence of multiple isoforms within the cell lines. GAPDH is used as a loading control. MW = molecular weight marker.

We therefore evaluated a subset of the target genes analyzed in Fig 4, covering multiple cellular processes, to determine whether their mRNA levels increased upon knockdown of p53^R172H^. p53 knockdown was performed in two additional p53^R172H^-derived cell lines and knockdown confirmed by immunoblot (Fig 5A). When added to the 2 knockdown lines previously characterized (Fig 3), this generated a panel of 4 knockdown cell lines, and paired controls, for analysis. We found that the levels of p21, Cldn1, and Rad51 were commonly increased in knockdown cells relative to paired control cells (Fig 5B). This suggests that mutant p53 suppressed their expression, potentially by functionally interfering with p63 and p73.

The results above suggest that p63 and p73 are inhibited by mutant p53. They additionally suggest that p63 and p73 are inhibited by an alternate mechanism in p53 null HCC cells. We therefore assessed the levels of p63 and p73 in p53^fl/fl^- and p53^R172H^-derived cell lines by immunoblotting. We found that p53^fl/fl^ cell lines expressed lower levels of the TA isoforms of p63 and p73 relative to p53^R172H^ cell lines (Fig 5C). We also observed relatively higher expression of ΔNp63 in p53^fl/fl^ cell lines (Fig 5C). Together, these data suggest that p63 and p73 are inhibited in p53 null HCC cells.

## Discussion

Point mutations in the gene encoding the p53 tumor suppressor protein are commonly found in hepatocellular carcinoma [6–9]. In addition, p53 gene mutation and loss of function is associated with tumor progression, poor differentiation status and poor prognosis in this malignancy [8,9]. Moreover, prior work has demonstrated that p53 mutants display gain-of-function properties in certain contexts [16–19]. Therefore, given the common occurrence of p53 mutations in HCC, we sought to determine whether a frequent tumor-associated mutation, R175H (R172H in mice) displays gain-of-function properties in HCC.

Consistent with gain-of-function properties, and in line with prior published studies, we found that mouse HCC cell lines isolated from tumors expressing the p53^R172H^ mutant depended on this mutant for their transformed properties. However, direct comparison of the phenotypes of HCC cell lines null for p53 and those expressing only mutant p53 demonstrated that the two classes of cell lines are phenotypically indistinguishable. Thus, while p53^R172H^ is required for the full transformation properties of HCC cells expressing this mutant protein, these properties are not greater than those observed in the p53-deficient setting.

These *in vitro* findings mirror our data from an HCC mouse model in which p53^R172H^ promotes HCC progression and metastasis, but only to a similar extent as p53 nullizygosity. These findings are in contrast to prior results suggesting that p53 mutants promote the increased development of carcinomas relative to p53 loss [20,21], as well as other data demonstrating the promotion of PDAC metastasis by mutant p53 [16]. However, they are in line with prior work demonstrating that p53 mutants display dominant-negative, but not gain-of-function properties during skin carcinogenesis [24]. A possible explanation for the differing results is that different p53 mutants display different properties in different tissues. For example, germline introduction of the p53^R270H^ mutant resulted in the development of carcinomas, including HCC, in p53 heterozygous mice, whereas the R172H mutant induced the development of osteosarcomas [20]. Therefore, it would be interesting to determine whether other p53 mutants such as R270H and R246S (corresponding to human hotspots R273H and R249S), display gain-of-function properties during HCC development *in vivo*.

It also remains to be seen whether the impact of p53 deficiency versus p53 mutation is dependent on the driving oncogenic lesion. Our mouse model is driven by the expression of the PyMT oncogene, which robustly activates signaling through the PI3K signaling axis. Indeed, our prior data demonstrate that abrogation of PyMT-induced activation of PI3K impairs HCC development [11]. Whether p53 nullizygosity and p53 mutation exert similar effects in liver tumors driven by the expression of commonly activated HCC oncogenes such as MYC and β-catenin remains to be tested.

Collectively, our results suggest that p53 mutation and p53 deletion result in hepatocellular tumors with similar functional impairments. Prior work has suggested that one potential mechanism through which p53 mutants exerted their gain-of-function effects is through the inhibition of the related transcription factors p63 and p73 [20,21,26]. Further, published experiments demonstrated that p53 mutants could inhibit p63 and p73 transcriptional activity in HCC cells [22]. We therefore examined the impact of shRNA-mediated p53^R172H^ silencing and found that the levels of p63- and p73-regulated genes were induced following knockdown of mutant p53. Yet, the levels of these target genes were similarly suppressed in p53 null HCC cells, suggesting that p63 and p73 activity were likewise impaired in these cells. In agreement, we observed lower TAp63 and TAp73 levels, and higher ΔNp63 levels in p53 null HCC cells relative to p53^R172H^-expressing HCC cells. Our data therefore suggest that p63 and p73 may be critical factors that constrain HCC development *in vivo*, and their functional inhibition is required for HCC development and progression. However, the mechanism(s) that impairs their activity in p53 null cells is currently unknown.

Of note, liver-specific inactivation of p63 or p73 has not been evaluated for its impact on liver carcinogenesis. These studies will be needed to determine whether p63 or p73 is the critical protein inactivated during liver tumor development. Perhaps an interesting clue comes from prior experiments performed by Jacks and colleagues. In their studies, combined inactivation of p53 and p73, but not p53 and p63, resulted in the development of spontaneous liver tumors *in vivo*, suggesting that p73 may play a more important role in constraining liver tumorigenesis than p63 [28]. In agreement, prior work has demonstrated that transgenic expression of the ΔNp73 isoform induces the formation of liver tumors *in vivo* [41]. However, studies directly comparing the tumor suppressive effects of the p63 and p73 transcription factors in the liver will be required before any conclusions can be drawn regarding their functions in this tissue.

## Supporting Information

S1 Fig p53^R172H^ tumors express mutant p53. A) Representative images of immunostaining for p53 in liver tumors induced in p53^fl/fl^ and p53^R172H^ mice. Nuclear p53 staining is observed in p53^R172H^ tumors but not p53^fl/fl^ tumors. Magnification 100x. B) Genomic DNA isolated from representative mouse liver tumors were examined by PCR for allelic recombination. In the top gel, the band representing 1 LoxP site demonstrates that recombination occurred at the Lox-Stop-Lox cassette in the *p53LSL-R172H* allele. The lower band, labeled “wild-type” represents the *Trp53* allele that does not contain an LSL cassette. The band on the lower gel represents recombination of the *p53* ^*flox*^ allele, demonstrating that deletion of exons 2–10 occurred.
(TIF)

S2 Fig HCCs induced in p53^R172H^ and p53^fl/fl^ livers display similar proliferation rates. A) Representative images of Ki67 IHC in normal liver tissue and HCC from *p53fl/fl* and *p53R172H/fl* mice from the survival cohort. B) Cells with Ki67-positive and negative nuclei were counted to obtain the percentage of cells within an HCC section that were Ki67 positive. Five fields in each tumor section were counted to obtain an average. In each cluster of bars, the first bar is the quantification of Ki67 staining in a non-tumor bearing liver.
(TIF)

S3 Fig HCCs induced in p53^R172H^ and p53^fl/fl^ livers metastasize to the lungs. Representative H&E images of lung metastases identified in mice bearing p53 null and p53^R172H^-expressing liver tumors.
(TIF)

S1 Table Primers for quantitative RT-PCR. (DOCX)

S2 Table Tumor and metastasis incidence in p53^fl/fl^ and p53^R172H^ mice. (DOCX)
