# Supplementary figures and images for: The p53R172H Mutant Does Not Enhance Hepatocellular Carcinoma Development and Progression
Source: PLoS One. 2015 Apr 17;10(4):e0123816. doi: 10.1371/journal.pone.0123816 (PMC4401698; doi:10.1371/journal.pone.0123816)

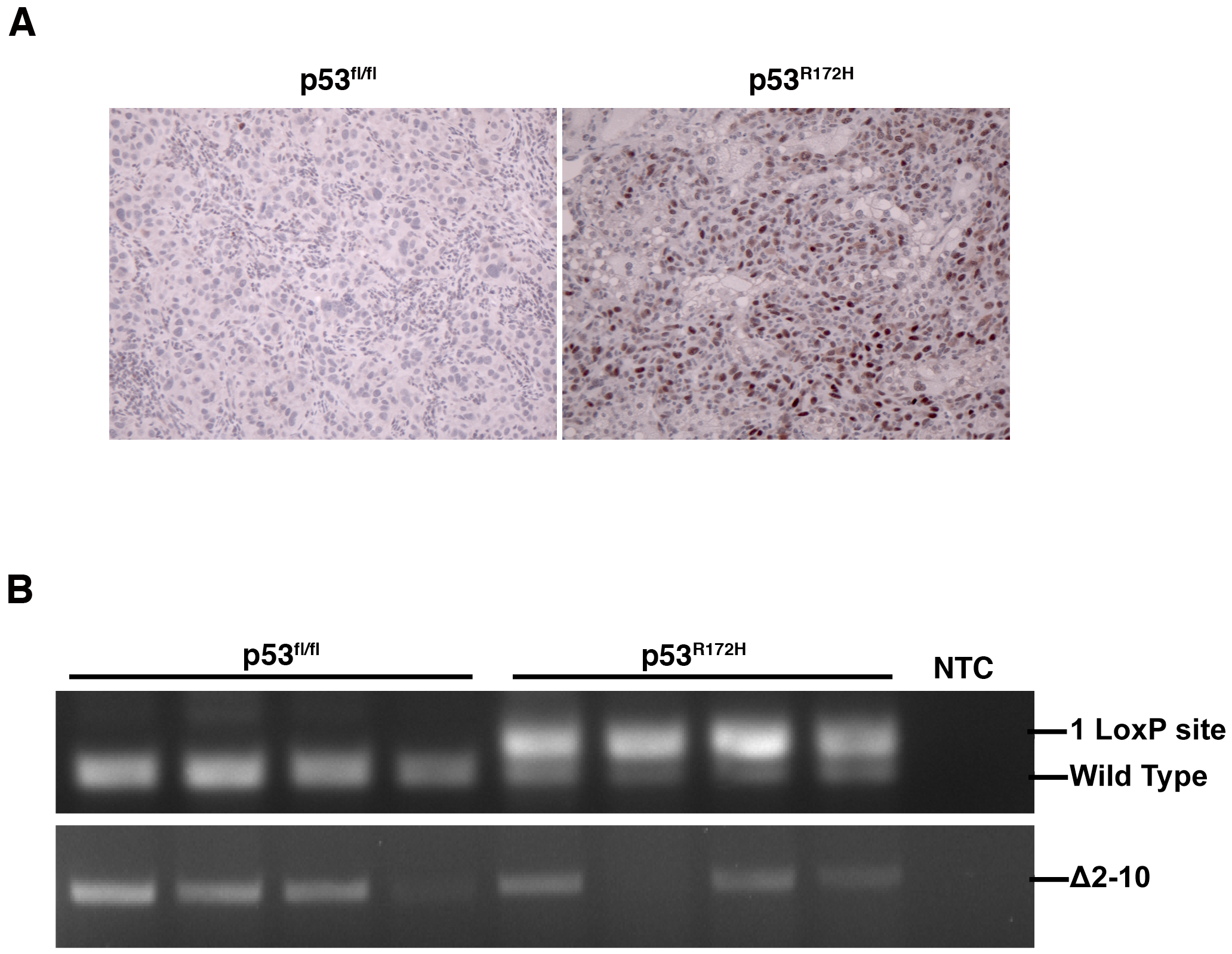

Supplement: S1 Fig — A) Representative images of immunostaining for p53 in liver tumors induced in p53fl/fl and p53R172H mice. Nuclear p53 staining is observed in p53R172H tumors but not p53fl/fl tumors. Magnification 100x. B) Genomic DNA isolated from representative mouse liver tumors were examined by PCR for allelic recombination. In the top gel, the band representing 1 LoxP site demonstrates that recombination occurred at the Lox-Stop-Lox cassette in the p53LSL-R172H allele. The lower band, labeled “wild-type” represents the Trp53 allele that does not contain an LSL cassette. The band on the lower gel represents recombination of the p53 flox allele, demonstrating that deletion of exons 2–10 occurred. (TIF) [file pone.0123816.s001.tif]

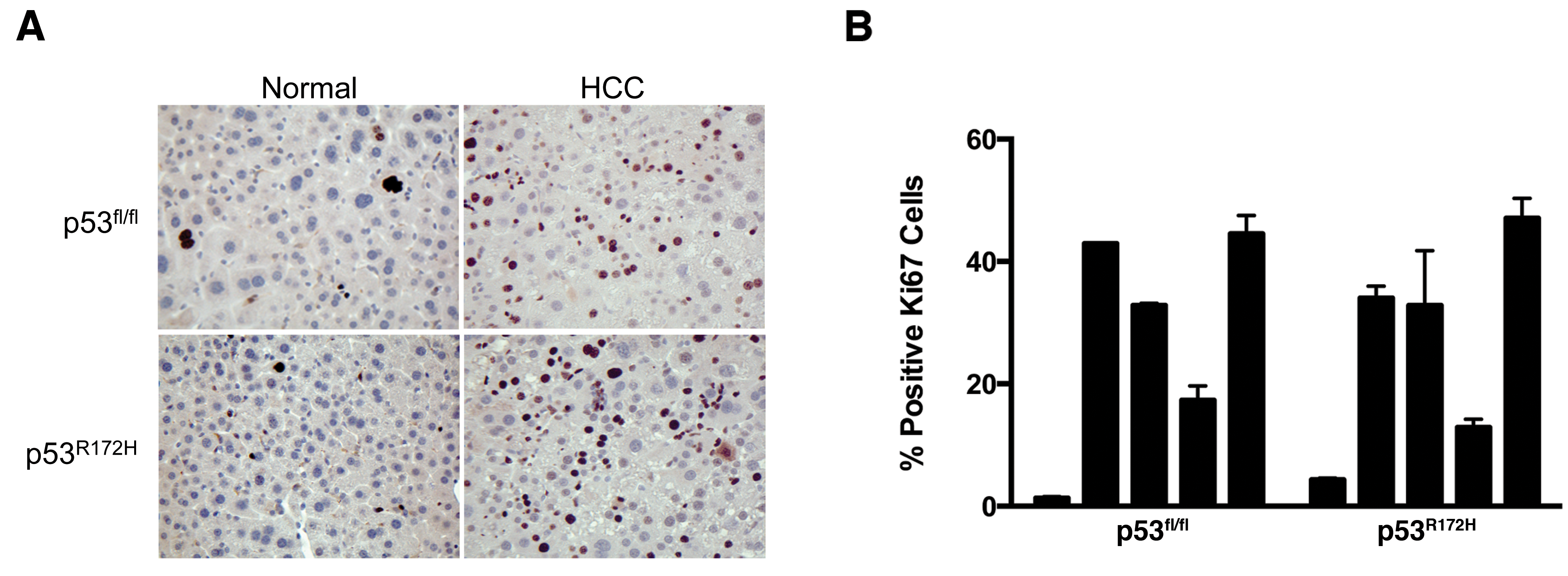

Supplement: S2 Fig — A) Representative images of Ki67 IHC in normal liver tissue and HCC from p53fl/fl and p53R172H/fl mice from the survival cohort. B) Cells with Ki67-positive and negative nuclei were counted to obtain the percentage of cells within an HCC section that were Ki67 positive. Five fields in each tumor section were counted to obtain an average. In each cluster of bars, the first bar is the quantification of Ki67 staining in a non-tumor bearing liver. (TIF) [file pone.0123816.s002.tif]

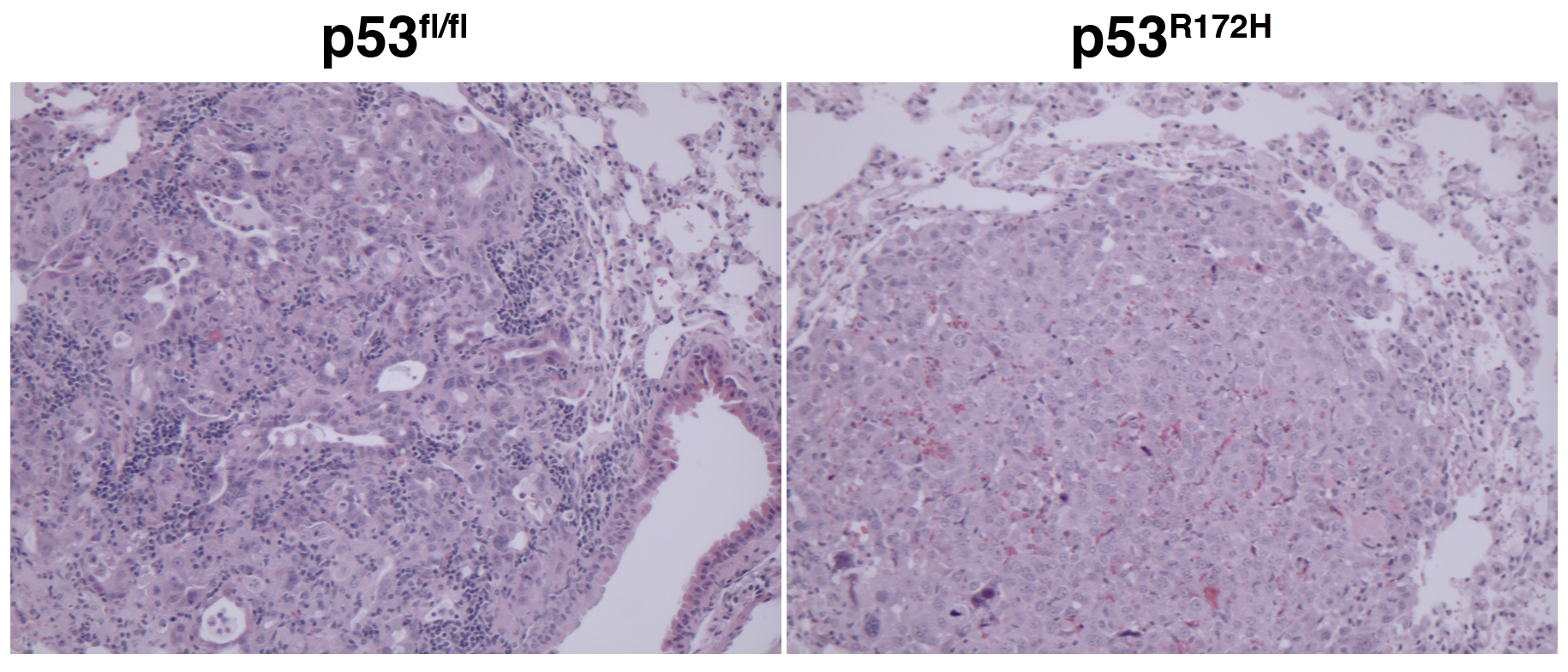

Supplement: S3 Fig — Representative H&E images of lung metastases identified in mice bearing p53 null and p53R172H-expressing liver tumors. (TIF) [file pone.0123816.s003.tif]
