# Supplementary material for: The p53R172H Mutant Does Not Enhance Hepatocellular Carcinoma Development and Progression
Source: PLoS One. 2015 Apr 17;10(4):e0123816. doi: 10.1371/journal.pone.0123816 (PMC4401698; doi:10.1371/journal.pone.0123816)
Supplement: S1 Table — (DOCX) [file pone.0123816.s004.docx]

**Table S1. Primers for quantitative RT-PCR.**

| **Gene** | **Forward Primer** | **Reverse Primer** |
| --- | --- | --- |
| β-Actin | tcctcctgagcgcaagtactct | cggactcatcgtactcctgctt |
| Brca2 | ggatggctcttcaggatcattcg | gtgcttttgaagtaccattgaccttta |
| Mre11 | cgaagaacgttgaaaggttcaaagc | gggaacgactggggaatcctca |
| Rad51 | tgggaagctcaaaaccatctcttaac | gtgtgtttctgtgaatgtatgcctaat |
| 14-3-3g | ttggacagtggttcgttcag | agcaactggtgcagaaagc |
| Egfr | ctgcaggctcagaaagtggt | acactgctggtgttgctgac |
| Itga3 | tgaggggacacaggtacaca | agactgagcgacaacagcg |
| Itga5 | gacagcaccaccttgcagta | ttctccgtggagttttaccg |
| Cldn1 | ctgcacagagagcaagggta | agtggcagatgcagaaagtg |
| Fn1 | actggatggggtgggaat | ggagtggcactgtcaacctc |
| Atg5 | aagtctgtccttccgcagtc | tgaagaaagttatctgggtagctca |
| Atg7 | atgccaggacaccctgtgaacttc | acatcattgcagaagtagcagcca |
| Ccng2 | ctgagaaatgccaaagtgga | gggccaagaatctatccaaa |
| Dcr1 | tgatgggaacgctaacacat | tctgctcagagtccatcctg |
| p21 | atcaccaggattggacatgg | cggtgtcagagtctagggga |
| Sharp1 | gacagccattgaacatggac | cttggtatcgtctcgcttca |
