# Supplementary material for: The p53R172H Mutant Does Not Enhance Hepatocellular Carcinoma Development and Progression
Source: PLoS One. 2015 Apr 17;10(4):e0123816. doi: 10.1371/journal.pone.0123816 (PMC4401698; doi:10.1371/journal.pone.0123816)
Supplement: S2 Table — (DOCX) [file pone.0123816.s005.docx]

**Table S2. Tumor and metastasis incidence in p53^fl/fl^ and p53^R172H^ mice.**

**p53^fl/fl^**

| **Age (weeks)** | **Tumor incidence (%)** | **Metastasis (%)** |
| --- | --- | --- |
| 12-16 | 9/16 (56) | 3/9 (33) |
| 17-24 | 6/8 (75) | 1/6 (17) |
| 25-35 | 9/16 (56) | 1/9 (11) |
| Total | 24/40 (60) | 5/24 (21) |

**p53^R172H^**

| **Age (weeks)** | **Tumor incidence (%)** | **Metastasis (%)** |
| --- | --- | --- |
| 12-16 | 4/13 (31) | 2/4 (50) |
| 17-24 | 6/8 (75) | 1/6 (17) |
| 25-35 | 8/12 (67) | 1/8 (13) |
| Total | 24/40 (60) | 5/24 (21) |
